# *FTO* at rs9939609, Food Responsiveness, Emotional Control and Symptoms of ADHD in Preschool Children

**DOI:** 10.1371/journal.pone.0049131

**Published:** 2012-11-14

**Authors:** Fleur P. Velders, Jolanda E. De Wit, Pauline W. Jansen, Vincent W. V. Jaddoe, Albert Hofman, Frank C. Verhulst, Henning Tiemeier

**Affiliations:** 1 Department of Child and Adolescent Psychiatry, Erasmus Medical Center – Sophia Children’s Hospital, Rotterdam, The Netherlands; 2 The Generation R Study Group, Erasmus MC University Medical Center, Rotterdam, The Netherlands; 3 Department of Epidemiology, Erasmus MC University Medical Center, Rotterdam, The Netherlands; 4 Department of Pediatrics, Erasmus Medical Center – Sophia Children’s Hospital, Rotterdam, The Netherlands; 5 Department of Psychiatrie, Erasmus Medical Center, Rotterdam, The Netherlands; University of Missouri-Kansas City, United States of America

## Abstract

The *FTO* minor allele at rs9939609 has been associated with body mass index (BMI: weight (kg)/height (m)^2^) in children from 5 years onwards, food intake, and eating behaviour. The high expression of *FTO* in the brain suggests that this gene may also be associated with behavioural phenotypes, such as impulsivity and control. We examined the effect of the *FTO* minor allele (A) at rs9939609 on eating behaviour, impulsivity and control in young children, thus before the BMI effect becomes apparent. This study was embedded in the Generation R Study, a population-based cohort from fetal life onwards. 1,718 children of European descent were genotyped for *FTO* at rs9939609. With logistic regression assuming an additive genetic model, we examined the association between the *FTO* minor allele and eating behaviour, impulsivity and control in preschool children. There was no relation between *FTO* at rs9939609 and child BMI at this age. The A allele at rs9939609 was associated with increased food responsiveness (OR 1.21, *p* = 0.03). Also, children with the A allele were less likely to have symptoms of ADHD (OR 0.74, *p* = 0.01) and showed more emotional control (OR 0.64, *p* = 0.01) compared to children without the A allele. Our findings suggest that before the association between *FTO* and BMI becomes apparent, the *FTO* minor allele at rs9939609 leads to increased food responsiveness, a decreased risk for symptoms of ADHD and better emotional control. Future studies are needed to investigate whether these findings represent one single mechanism or reflect pleiotropic effects of *FTO*.

## Introduction

Single nucleotide polymorphisms (SNPs) in intron 1 of the fat and obesity-associated transcript gene (*FTO*) in adults have consistently been associated with increased body mass index (BMI; weight (kg)/height (m)^2^) and obesity [1], [2]. In children the direction of the association between variance in *FTO* and BMI is age-dependent. In a large meta-analysis [3], the *FTO* rs9939609 minor allele was related to a lower body weight before the age of 2.5 years as compared to the body weight of carriers of the common allele. The relation between the *FTO* minor allele and a higher BMI as observed in adults became only evident in children after the age of 5.5 years.

The biological mechanism responsible for the association between *FTO* and obesity remains to be determined. Animal studies showed that *Fto* genotypes in mice are associated with increased body weight, fat mass and food intake [4]. In children aged 4–11 years, variation in *FTO* at rs9939609 has been associated with increased food intake independent of body weight [5], [6]. In 131 children aged 4–5 years, the *FTO* minor allele at rs9939609 (A) was associated with higher consumption of highly palatable food [7]. In a large sample of children aged 7–13 years, the AA genotype was associated with reduced satiety responsiveness [8]. Furthermore, children (6–19 years) with the *FTO* minor allele at rs9939609 were more likely to report loss of control over eating [9]. To the best of our knowledge, a possible effect of *FTO* on behavioural problems, such as impulsivity and other aspects of executive functioning, has not been studied yet.

*FTO* is highly conserved and widely expressed in both central and peripheral tissues [10]. The high expression of *FTO* in the hypothalamus, which is known to be involved in the control of energy homeostasis, possibly underlies the association between *FTO*, food intake and body weight [11]. *FTO* is also highly expressed in other brain areas such as the cortex, the hippocampus and the cerebellum. Hence, it has been suggested that *FTO* is involved in other functions as well [2], [10]. This has been confirmed by reports showing an effect of *FTO* on overall-mortality, especially with an increased risk for diseases of the nervous system, which was independent of BMI [12]. Variation in *FTO* was also related to reduced brain volume in the healthy elderly [13].

In light of evidence for high expression of *FTO* in the brain and its relation with eating behaviour, the association of genetic variation in *FTO* with child behavioural phenotypes merits further investigation. Albeit inconsistently, child obesity has been associated with uncontrolled eating behaviour, and with emotional and behavioural problems such as impulsivity and attention-deficit/hyperactivity disorder (ADHD) [14], [15]. In the study by Waring and Lapane, the risk of child overweight was larger in unmedicated children with ADHD, whereas the risk of underweight was larger in medicated children with ADHD [16]. Lawlor and colleagues reported an inverse association between BMI and psychological distress, in which common variance in *FTO* was used as instrumental variable [17]. Terracciano and colleagues reported an association between impulsivity and overweight, which was independent of *FTO* status. This suggests that *FTO* does not necessarily account for the association between impulsivity and overweight [18].

Our aim was to investigate the role of *FTO* at rs9939609 in eating behaviour, impulsivity and control in preschool children participating in a large population-based cohort. At this young age, no effect of common variance of *FTO* on BMI can be expected [3]. We hypothesized that the association between *FTO* at rs9939609 and eating behaviour may already be found in preschool children and possibly precedes the association between *FTO* and BMI. Given recent findings by Lawlor et al and Terracciano et al, *FTO* may not account for the association between BMI and behaviour, but instead may have an independent relation to behavioural phenotypes.

These hypotheses were tested in preschool children participating in a large population-based cohort. We explored the association between *FTO* at rs9939609, symptoms of ADHD and symptoms of Oppositional Defiant Disorder (ODD) at the age of 3 years. ADHD and ODD are both associated with impulsivity and the comorbidity of these disorders may largely be explained by shared genetic variance [19]. So we tested for the effect of the *FTO* minor allele on the risk of symptoms of ADHD and ODD to explore whether this effect was specific to ADHD or not. To study specific cognitive aspects of impulsive behaviour, we also examined the association between *FTO* at rs9939609 and components of executive functioning at the age of 4 years.

## Subjects and Methods

### Design

This study was embedded in the Generation R Study, a population-based cohort from foetal life onwards in Rotterdam, the Netherlands. The Generation R Study is a prospective population-based cohort study from fetal life onwards in the city of Rotterdam, the Netherlands. It was designed to identify early biological and environmental determinants of growth, development and health in fetal life and childhood. The Generation R Study has previously been described in detail [20], [21] In short, all pregnant women living in the study area with a delivery date between April 2002 and January 2006 were eligible for enrollment in the Generation R Study. In total, 9,778 pregnant women were included, of whom 8,880 enrolled in the prenatal part of the study. The participating women gave birth to 9,745 live born children. Due to exclusion of participants in the pilot phase (12%) and because of withdrawal from the study (7%), 7,893 children participated in the postnatal phase of the Generation R Study. The study has been approved by the Medical Ethics Committee of the Erasmus Medical Centre, Rotterdam. Written informed consent was obtained from parents of the participating children.

### Population of Analysis

This study was restricted to children of Northern European descent, which was determined by principle component analyses of genome wide association data, as described previously [20]. Principle component analyses yield factors that can be interpreted as the direction which maximizes the variance of the sample while being uncorrelated to previous components. Within the children of European descent (n = 2650), *FTO* rs9939609 information was available in 2557 children. In 1718 (67%) of these children, information was available about child BMI, problem behaviour and eating behaviour. These 1718 children comprised the population of analysis.

### Genotyping

DNA was collected from cord blood at birth. Participants were genotyped for *FTO* rs9939609. Genotyping was performed using Taqman allelic discrimination assay (Applied Biosystems, Foster City, CA) and Abgene QPCR ROX mix (Abgene, Hamburg, Germany). The genotyping reaction was amplified using the GeneAmp® PCR system 9600 (95°C for 15 minutes, then 40 cycles of 94°C for 15 seconds and 60°C for 1 minute). The fluorescence was detected on the 7900HT Fast Real-Time PCR System (Applied Biosystems) and individual genotypes were determined using SDS software (version 2.3, Applied Biosystems). Genotyping was successful in 97–99% of the samples. To confirm the accuracy of the genotyping 276 randomly selected samples were genotyped for a second time with the same method. The error rate was less than 1% for all genotypes. To check for potential contamination with maternal blood, sex was determined in male participants. Contamination occurred in <1% of cases. Allele frequencies adhered to Hardy Weinberg Equilibrium (HWE) (*p = *0.405).

### Child Eating Behaviour

At the age of four years, eating behaviour was assessed using the Child Eating Behavior Questionnaire (CEBQ). The CEBQ [22] is designed to assess variation in eating style among children. In this 35-item instrument, parents rate their child’s eating behaviour during the past month on a five-point Likert scale (1 = never to 5 = always). The CEBQ consists of four subscales that measure food approach behaviours: Emotional Overeating, Enjoyment of Food, Food Responsiveness, and Desire to Drink and three subscales that quantify food-avoidant responses: Emotional Undereating, Satiety Responsiveness, and Fussiness. Examples of items are “My child loves food” (Enjoyment of Food), “Even if my child is full, he finds room to eat his favourite food” (Food Responsiveness), and “My child has a big appetite” (Satiety Responsiveness). The CEBQ data has good psychometric properties, such as concurrent validity with actual eating behaviour, test-retest reliability, and stability over time [23].

The Cronbach’s alpha of all items was 0.99. Genetic variation in the *FTO* gene has previously been associated with increased food intake and satiety responsiveness. Therefore, the scales related to food approach behaviours (Emotional Overeating, Enjoyment of Food, Food Responsiveness) and one subscale about food avoidance (Satiety Responsiveness) were selected to examine the effect of FTO and food related behaviour in our sample. The alpha’s of these subscales ranged from 0.78–0.89.The CEBQ scales could not be normalized and were analyzed as dichotomized variables with a cut off at the highest 20 percent of the item scores in line with the CBCL cut-off.

### Child Behaviour

The Child Behaviour Checklist/1½–5 (CBCL/1½–5) was used to obtain standardized parent reports of children’s emotional and behavioural problems at the age of 3 years. This questionnaire contains 99 items, which are scored on a three-point scale: 0 = not true, 1 = somewhat true or sometimes true, and 2 = very true or often true, based on the two preceding months. The CBCL/1½–5 comprises 5 DSM-oriented Scales; Affective Problems, Anxiety Problems, Pervasive Developmental Problems, Attention Deficit/Hyperactivity Problems and Oppositional Defiant Problems. The psychometric properties of the CBCL are well established [24]. The Cronbach’s alpha based on the 99 items was 0.92. In this study we used the DSM-oriented scales Attention Deficit/Hyperactivity Problems (ADHD) and Oppositional Defiant Problems (ODD), which comprise problems with impulsivity and control. The alpha’s of ADHD was 0.75 and 0.64 for ODD. These scales could not be normalized and were analyzed as dichotomous variables. In the absence of Dutch norm scores, we defined a non-optimal score as the highest 20 percent of the item scores in line with previous studies [25].

### Child Executive Functioning

The Behavior Rating Inventory of Executive Function-Preschool Version (BRIEF-P) was used to assess child executive function at 4 years. The BRIEF-P is a questionnaire for parents to assess the executive function behaviour in a broad age range of preschoolers (2–5 years) [26], [27]. It contains 63 items within five related clinical scales that measure different aspects of executive functioning: emotional control, inhibit, shift, working memory, and plan/organize. The parents were asked to rate problematic behaviour of the child in the preceding month on a three-point scale (never, sometimes, and often). In the present study, we used the clinical scales emotional control, inhibit and shift as in general young children show most problems on these components of ADHD. The Cronbach’s alpha of all 63 items was 0.94. The alpha’s of the clinical scales were; “inhibit” 0.88, “shift” 0.81, “emotional control” 0.82. Based on sex and age, the raw scores of the clinical scales are transformed into *T* scores. A *T* score of 65 represents 1.5 standard deviations above the mean, and distinguishes non-clinical scores from clinical scores.

### Covariates

Gestational age was established by fetal ultrasound examinations. Information about birth weight and gender was obtained from midwife and hospital registries at birth; information about maternal age, marital status, parity and educational level was obtained by questionnaire. The highest completed education (primary school, secondary school or higher education) determined the educational level. At the age of three and four years, trained staff in the community health centers obtained children’s weight and length using standardized procedures. Child body mass index (BMI; kg/m^2^) at 3 and 4 years was calculated and sex and age adjusted standard deviation scores (SDS) of the Dutch reference growth curves were obtained (Growth Analyser 3.0, Dutch Growth Research Foundation). Also, a BMI sd of 1.1 or higher was used to define overweight or obesity [28], [29].These covariates were not selected to adjust for confounding, but to describe the study sample.

### Statistical Analysis

We examined selective non-response by comparing characteristics between mothers and children included (n = 1718) and those excluded (n = 839) from this study using chi-square statistics for categorical variables, independent t-tests for normally distributed continuous variables and Mann Whitney U-tests for non-normally distributed continuous variables. Using the same tests, we compared characteristics of mothers and children in this study by *FTO* genotype. The correlation between the outcome variables (symptoms of ADHD and ODD, executive function and eating behaviour), was calculated with the Spearman’s correlation coefficient for non-parametric variables (two-tailed). Logistic regression analyses were performed to test the association between *FTO* rs9939609 and child eating behaviour at 4 years. The analyses were run under the assumption of an additive genetic model, which also optimizes the power. In addition, we also present results for a three-categorical genetic model with the TT genotype as reference category to explore recessive or dominant effects. Logistic regression was also used to test for an association between *FTO* at rs9939609 and symptoms of ADHD and ODD at 3 years, and emotional control, inhibition and shift at 4 years. The level of significance for all analyses was set at α = 0.05. All statistical analyses were carried out using PASW Statistics, version 17.0 for Windows [30].

### Non-response Analysis

Children excluded from our study (n = 839) had on average younger (30.5 vs. 32.1 years, *t* = 9.23, *p*<0.001) and less highly educated mothers (28.5 vs. 43.0%, χ2 = 52.43(1 df), *p*<0.001), and had a lower birth weight (3503 versus 3573 g, t = 3.30, *p = *0.001) than children included in our study (n = 1718). The distribution of sex, birth order, BMI and the minor allele frequency of rs9939609 (61.4 vs. 60.6%, χ2 = 0.147(1 df), *p = *0.70) did not differ amongst these two groups.

## Results

The mother and child characteristics are presented in table 1. The distribution of the characteristics did not differ between children homozygous for the T allele (TT), heterozygous children (AT) or children homozygous for the A allele (AA) (table 1). Child BMI did not differ according to the *FTO* genotype at rs9939609.

**Table 1:** Sample characteristics by *FTO* rs9939609 genotype (n = 1718).

	Child *FTO* rs9939609			
	TT(n = 677)	AT(n = 790)	AA(n = 251)	*p* value
*Mother* a,c				
Age at child birth (years)	32.1(3.8)	32.2(4.1)	32.1(3.3)	0.99
Education				
higher education (%)	42.3	44.5	41.1	0.52
Marital status				
married/living together %	96.2	96.2	97.9	0.42
*Child* a,c				
Gestational age (weeks) b	40.4(32.7;43.0)	40.4(29.9;43.4)	40.3(35.4;43.0)	0.67
Birth weight (gram)	3575(506)	3566(495)	3589(530)	0.82
Birth order				
first child (%)	60.6	59.7	61.0	0.91
Gender				
boys (%)	48.4	51.8	55.0	0.17
Child age				
3 years assessment	36.47(0.04)	36.48 (0.04)	36.48 (0.07)	0.98
4 years assessment	468.42 (0.03)	48.53 (0.04)	48.39 (0.05)	0.05
BMI sd_score				
BMI age 3	0.25(0.99)	0.21(0.92)	0.24(0.98)	0.68
BMI age 4	0.12(0.90)	0.08(0.91)	0.16(0.88)	0.51
Overweight or obesity (%)^d^	10.3	11.1	12.4	0.68

a mean (standard deviation) unless otherwise indicated.

b median (100% range).

c with the chi-square statistic for categorical variables, one-way ANOVA for normally distributed continuous variables and the Kruskal-Wallis test for non-normally distributed.

continuous variables.

d overweight or obesity is defined as a BMI-sds >1.10.

Table 2 presents the correlation between the outcome measurements. It shows that symptoms of ADHD and ODD were positively correlated with emotional control and inhibition. Symptoms of ADHD and ODD were not significantly correlated with food approach, and symptoms of ADHD weakly correlated with satiety responsiveness (food avoidance) (Spearman’s rho 0.06, *p* value <0.01). Child BMI was positively correlated to food responsiveness and enjoyment of food. There was a negative correlation between child BMI and satiety responsiveness. Child BMI was not significantly correlated to the other behavioural phenotypes.

**Table 2:** Correlation between child BMI, child emotional and behavioural problems, executive function and eating behaviour.

	BMI	Symptomsof ADHD		Symptoms of ODD	Emotional control	Inhibition	Shift	Food response	Enjoyment of food	Emotional overeating	Satiety responsiveness
			n = 1717	n = 1714	n = 1638	n = 1621	n = 1636	n = 1718	n = 1718	n = 1718	n = 1718
BMI	–		−0.01	0.03	−0.02	0.02	−0.02	0.23***	0.06**	0.02	−0.02***
Symptoms of ADHD			–	0.35**	0.21**	0.31**	0.04	0.04	0.01	0.04	0.06*
Symptoms of ODD				–	0.27**	0.23**	0.08**	0.02	−0.03	0.01	0.01
Emotional control					–	0.31**	0.30**	0.05	−0.04	0.05	0.03
Inhibition						–	0.13**	0.03	0.01	0.06	0.06*
Shift							–	0.003	0.01	0.03	0.002
Food responsiveness								–	0.32	0.33**	0.09**
Enjoyment of food									–	0.29**	0.19**
Emotional overeating										–	0.27**
Satiety responsiveness											–

Spearman correlation coefficient (two tailed).

* <0.05, **<0.01 ***<0.001.

Table 3 presents the association between the *FTO* minor allele at rs9939609 and child eating behaviour at 4 years. The minor allele was associated with food responsiveness (OR 1.21, 95%CI 1.02;1.43, *p* value = 0.026), which did not materially change after adjustment for symptoms of ADHD (OR 1.22, 95%CI 1.04;1.45, *p* value = 0.018). This effect was slightly attenuated after adjustment for gender and child age (OR 1.17, 95%CI 0.98;1.40, *p* value = 0.09). However, child age and gender were not significantly associated with food responsiveness. Children with 2 copies of the minor allele (AA) were more likely to score high on food responsiveness (OR 1.45, 95%CI 1.03;2.05, *p* value = 0.04) compared to children without the minor allele. Children with the minor allele did not have an increased likelihood for high scores on enjoyment of food (OR 1.08, 95%CI 0.88;1.34, *p* value = 0.46) or emotional eating (OR 1.10, 95% CI 0.96;1.40, *p* value = 0.13). Neither was the minor allele related to satiety responsiveness (OR 1.05, 95%CI 0.89;1.24, *p* value = 0.56). Children with high scores on food responsiveness (24% versus 7.6%, χ2 = 77.8(1 df), *p<*0.001) and children with high scores on enjoyment of food (16.4% versus 10.6%, χ2 = 6.8(1 df), *p = *0.009) were more likely to be overweight/obese compared to children with low scores on these phenotypes. The difference in percentage overweight/obesity in children with high scores on satiety responsiveness compared to children with low scores did not reach significance (8.3% versus 11.6%, χ2 = 2.8(1 df), *p = *0.09 (see Supplementary Material Table S1).

**Table 3:** Associations of child *FTO* at rs9939609 with child eating behaviour at 4 years (n = 1718).

	Food responsiveness			Enjoyment of Food			Emotional overeating			Satiety responsiveness		
*FTO* at rs9939609	OR^a^	95%CI	*p*	OR^a^	95%CI	*p*	OR^a^	95%CI	*p*	OR^a^	95%CI	*p*
additive model	1.21	1.02;1.43	0.026	1.08	0.88;1.34	0.46	1.16	0.96;1.40	0.13	1.05	0.89;1.25	0.56
per genotype												
TT	ref			ref			ref			ref		
AT	1.23	0.95;1.59	0.12	1.22	0.88;1.68	0.23	1.17	0.87;1.56	0.29	0.97	0.75;1.27	0.84
AA	1.45	1.03;2.05	0.04	1.10	0.70;1.74	0.69	1.34	0.90;1.98	0.15	1.16	0.81;1.66	0.42

Abbreviations: OR, odds ratio; 95%CI, 95% confidence interval; ref, reference.

a The ORs represent the increased risk of high scores on CEBQ subscales per *FTO* minor allele at rs9939609.

In table 4, we present the association between the *FTO* minor allele at rs9939609 and symptoms of ADHD and ODD. The minor allele was associated with a lower risk for symptoms of ADHD (OR 0.74, 95%CI 0.59; 0.93, *p* value = 0.009), which was not attenuated by adjustment for child age and gender (OR 0.74, 95%CI 0.59; 0.93, *p* value = 0.008). Children with high scores on symptoms of ADHD had slightly higher birth weight than children with low ADHD symptoms. However, birth weight did not confound the association between *FTO* and ADHD. Preschool children with 2 *FTO* minor alleles (AA) were less likely to show symptoms of ADHD (OR 0.53, *p* value = 0.02) compared with children without the minor allele. The minor allele was not significantly associated with symptoms of ODD (OR 1.08, 95%CI 0.88; 1.34, *p* value = 0.46). The prevalence of overweight/obesity did not differ between children with high scores and low scores on ADHD an ODD symptoms (see SM table 1).

**Table 4:** Associations of child *FTO* at rs9939609 with behavioural problems at 3 years (n = 1718).

	Symptoms of ADHD			Symptoms of ODD		
*FTO* at rs9939609	OR^a^	95%CI	*p*	OR^a^	95%CI	*p*
additive model	0.74	0.59;0.93	0.009	1.08	0.88;1.34	0.46
per genotype						
TT	ref			ref		
AT	0.77	0.56;1.05	0.10	1.53	1.11;2.12	0.01
AA	0.53	0.32;0.89	0.02	0.93	0.57;1.54	0.78

Abbreviations: ADHD, attention deficit hyperactivity disorder; ODD, oppositional defiant disorder; OR, odds ratio; 95%CI, 95% confidence interval; ref, reference.

a The ORs represent the increased risk of high scores on CBCL DSM-oriented scales per *FTO* minor allele at rs9939609.

Table 5 presents the association between the *FTO* minor allele at rs9939609 and executive function. The minor allele was associated with less problems with emotional control (OR 0.64, 95%CI 0.47;0.88, *p* value = 0.006), which was not attenuated by adjustment for symptoms of ADHD (OR 0.69, 95%CI 0.50;0.96, *p* value = 0.026) or child age and gender (OR 0.61, 95%CI 0.44;0.84, *p* value = 0.002). Children with problems in executive function had similar birth weight than children without executive function problems. Children with the AA genotype were significantly less likely to have problems with emotional control (OR 0.31, 95%CI 0.13;0.74, *p* value = 0.01) compared to children without the minor allele. The association between the *FTO* minor allele, and problems with Inhibit and Shift did not reach significance. The prevalence of overweight/obesity did not differ between children with high scores and low scores on these components of executive functioning (see Table S1).

**Table 5:** Associations of child *FTO* at rs9939609 with child executive function at 4 years.

	problems with Emotional control			problems with Inhibition			problems with Shift		
	(n = 1637)			(n = 1620)			(n = 1636)		
***FTO*** ** at rs9939609**	OR^a^	95%CI	*p*	OR^a^	95%CI	*p*	OR^a^	95%CI	*p*
additive model	0.64	0.47;0.88	0.006	0.81	0.57;1.16	0.26	0.79	0.58;1.09	0.15
**per genotype**	**OR** a	**95%CI**	***p***	**OR** a	**95%CI**	***p***	**OR** a	**95%CI**	***P***
TT	ref			ref			ref		
AT	0.75	0.50;1.15	0.18	0.73	0.43;1.22	0.22	0.84	0.54;1.32	0.45
AA	0.31	0.13;0.74	0.01	0.73	0.34;1.55	0.41	0.59	0.28;1.22	0.15

Abbreviations: OR, odds ratio; 95%CI, 95% confidence interval; ref, reference.

a The ORs represent the increased risk of clinical scores on BRIEF-P Emotional Control and Inhibition per *FTO* minor allele at rs9939609.

## Discussion

In a large population-based cohort, children with 2 copies of the *FTO* minor allele (A) at rs9939609 were less likely to have symptoms of ADHD and had less problems with emotional self-control compared with the other children. Independent of symptoms of ADHD, the *FTO* minor allele was associated with increased food responsiveness.

First, we explored the association between *FTO*, preschool BMI and eating behaviour. As expected at this young age, there was no significant association between the *FTO* minor allele at rs9939609 and child BMI. At preschool age, children with high scores on food responsiveness and enjoyment of food already had a higher BMI compared to low scoring children. Even in these young children, the *FTO* minor allele was already associated with increased food responsiveness. The direction of the effect of the minor allele on eating behaviour in our study is similar to previous reports about *FTO* and behaviour related to food approach. As designed by Wardle and colleagues, the scale “food responsiveness” detects levels of maladaptive appetite and assesses the tendency to eat when prompted by external cues [23]. It seems plausible that this type of eating behaviour accounts at least partially for the relation of *FTO* with increased food intake. The association of the minor allele at rs9939609 with food responsiveness was not altered by adjustment for child symptoms of ADHD, which indicates that the effect of this minor allele on eating behaviour was not explained by the association between *FTO* and symptoms of ADHD.

Second, we tested for an effect of the *FTO* minor allele at rs9939609 on symptoms of ADHD and ODD. Independent of eating behaviour, the *FTO* minor allele was associated with a decreased likelihood of symptoms of ADHD. Possibly, this effect is specific to certain phenotypes of ADHD, as no association with ODD was found, which is often co-morbid to some forms of ADHD. Most plausibly, the association between the AT genotype and ODD is false-positive, since we had no hypothesis on prior data to suggest that heterozygosity at rs9939609 is associated with negative or positive outcomes. Recently, two studies used common variance in *FTO* as instrumental variable in mendelian randomization designs to examine the association between BMI and psychopathology in adults. In a cohort of London-based civil servants, variance in *FTO* was associated with long-term obesity and independently with common mental disorders [31]. However, this association was found in men only and with a different variant of *FTO* (rs1421085) than investigated in the current study. In a study including more than 50,000 subjects, the mendelian randomization design showed an inverse association between common variance in *FTO*, BMI, and psychological distress [17]. In line with this inverse association reported by Lawlor and colleagues, we reported an inverse association between *FTO* at rs9939609 and symptoms of ADHD. In our study, child BMI did not differ according to low or high scores on symptoms of ADHD. This may be partly explained by parental control over eating in these young children, which are on average also fairly active. It seems less likely that the use of stimulant medication influenced this association, since Dutch children at this age are not yet diagnosed with ADHD and unlikely to receive stimulant medication. This could also indicate that the association between BMI and ADHD as observed in previous studies may not be further explained by the minor allele of *FTO* at 9939609. Terraccino and colleagues reported that in persons 14 to 94 years of age impulsivity was associated with higher BMI, independent of *FTO* status. An association between *FTO* and impulsivity was, however, not tested.

In young children, ADHD is characterized by temperamental problems in self-regulation, shown by inattentiveness, overactivity and impulsiveness [32]. Hence, to test for the effect of FTO on specific cognitive aspects of ADHD, we examined the association between the minor allele of FTO, and emotional control, inhibition and shift at the age of 4 years. We were able to show that the minor allele of FTO at rs9939609 is associated with better emotional control, independent of symptoms of ADHD. The work of Sovio and colleagues may help explain these findings. They found a relation between the minor allele at rs9939609 and the age of the adiposity rebound in young children [3]. The adiposity rebound marks the transition from a decline in BMI to a rise in BMI, which on average occurs around the age of 5 to 6 years. An earlier adiposity rebound has been associated with obesity and accelerated growth [33]. Hence, it was posited that carriers of this minor allele might be more developmentally advanced than wild type carriers [3]. Our findings may support this theory, since children differed in their ability to control their behaviour according to the minor allele of *FTO* at rs9939609. A child’s ability to control its behaviour is expected to improve with age and thus reflects a stage of development.

Alternatively, our findings may be explained by specific behaviour of children with the minor allele of *FTO*. Compared to non-carriers, these children tend to respond more to highly palatable food, which is known to stimulate dopamine pathways [34]. These dopamine pathways are thought to be involved in the pathophysiology of ADHD [35]. Hence, the eating behaviour of children with the *FTO* minor allele may reduce symptoms of ADHD by acting as a natural reward. Therefore, the relation between the *FTO* minor allele at rs9939609 and less symptoms of ADHD might actually be the result of specific eating habits, which one can view as “self-medication by eating”. Finally, our findings may reflect pleiotrophy of *FTO*. Pleiotrophy refers to the effect of one genetic region on more than one phenotype, which is the result of a single gene that can be transcribed differently or a single gene product that can affect multiple phenotypes [36]. Clearly, further imaging and biomedical research is warranted to reveal the mechanism underlying these effects of *FTO*.

This study has considerable strengths, such as the large sample size and the minimization of possible bias of population heterogeneity by using GWAS data to select children of Northern European descent. Despite these strengths, several limitations should be discussed. First, the restriction to children of Northern European descent not only minimizes the bias by population heterogeneity, it also limits the generalizability of our findings to other populations. Second, observational measurements in this large cohort were not feasible. Therefore, we relied on maternal report of child behaviour, executive function and eating behaviour. Yet, we used validated questionnaires with good reliability and validity. Third, symptoms of ADHD and ODD were studied rather than clinical diagnoses based on the DSM IV classification. Also, information about the use of stimulant medication was not available in our sample. Thus, future studies are needed to confirm our results in clinical populations. Fourth, the level of children’s physical activity may influence the association between the FTO minor allele and BMI. Ruiz and colleagues showed that the FTO minor allele was associated with a higher BMI in less active adolescents only, and not in active adolescents [37]. Possibly, the association between the FTO minor allele and child behaviour is also influenced by physical activity. However, in our sample this information was not available at the age of 3 or 4, and thus this interaction could not be tested.

Overall, this study provides initial support for an association of the *FTO* minor allele at rs9939609 with decreased risk for symptoms of ADHD and less problems with emotional control in preschool children. Independent of symptoms of ADHD, *FTO* was also related to food responsiveness. Future research is needed to determine whether these findings can be explained by a single underlying mechanism such as accelerated development or self-medication by eating, or that they reflect the pleiotrophy of the *FTO* genotype.

## Supporting Information

Table S1 Percentage of overweight/obesity in groups of children with low scores versus high scores on behavioural phenotypes.
(DOC)
