# Supplementary material for: FTO at rs9939609, Food Responsiveness, Emotional Control and Symptoms of ADHD in Preschool Children
Source: PLoS One. 2012 Nov 14;7(11):e49131. doi: 10.1371/journal.pone.0049131 (PMC3498333; doi:10.1371/journal.pone.0049131)
Supplement: Table S1 — Percentage of overweight/obesity in groups of children with low scores versus high scores on behavioural phenotypes. (DOC) [file pone.0049131.s001.doc]

**PONE-D-12-04584**

**FTO at rs9939609, Food Responsiveness, Emotional Control and Symptoms of**

**ADHD in Preschool Children**

**PLoS ONE**

Supplementary Material Table S1

| Table S1 Percentage of overweight/obesity in groups of children with low scores versus high scores on behavioural phenotypes. | | | | |
| --- | --- | --- | --- | --- |
|  | Percentage of  Overweight/obesity^b^ | | Chi- square | *p* value |
| Behavioural problem score | low^a^ | high^a^ |  |  |
| Symptoms of ADHD | 11.2 | 9.8 | 0.33 | 0.57 |
| Symptoms of ODD | 10.7 | 13.4 | 1.34 | 0.25 |
| Executive functioning |  |  |  |  |
| Emotional control | 11.1 | 8.0 | 0.90 | 0.34 |
| Inhibition | 10.8 | 12.9 | 0.30 | 0.58 |
| Shift | 11.1 | 7.8 | 0.95 | 0.33 |
| Eating behaviour |  |  |  |  |
| Food responsiveness | 7.6 | 24.0 | 77.8 | <0.001 |
| Enjoyment of food | 10.6 | 16.4 | 6.82 | 0.009 |
| Emotional overeating | 11.0 | 11.1 | 0.004 | 0.95 |
| Satiety responsiveness | 11.6 | 8.3 | 2.97 | 0.09 |
| ^a^ low symptoms scores are defined by scores < 80^th^ percentile, high symptoms scores are defined by scores ≥ 80^th^ percentile.  ^b^ overweight/obesity is defined by a BMI sd score > 1.10 | | | | |
